# Improved outcome in children with advanced stage B-cell non-Hodgkin's lymphoma (B-NHL): results of the United Kingdom Children Cancer Study Group (UKCCSG) 9002 protocol

**DOI:** 10.1054/bjoc.1999.1083

**Published:** 2000-03-21

**Authors:** A Atra, J D Imeson, R Hobson, M Gerrard, I M Hann, O B Eden, R L Carter, C R Pinkerton

**Affiliations:** Department of Paediatric Oncology, The Royal Marsden Hospital NHS Trust, Downs Road, Sutton, Surrey, SM2 5PT, UK; UKCCSG, Department of Epidemiology and Public Health, University of Leicester, 22–28 Princess Road West, Leicester, LE1 6TP, UK; Sheffield Children's Hospital NHS Trust, Western Bank, Sheffield, S10 2TH, UK; The Hospital For Sick Children, Great Ormond Street, London, WC1 3JH, UK; Academic Unit of Paediatric Oncology, Christie Hospital NHS Trust, Manchester, M20 9BX, UK

**Keywords:** childhood, B-cell non-Hodgkin's lymphoma, advanced stage III and IV, UKCCSG 9002 protocol

## Abstract

From July 1990 to March 1996, 112 children with stage III or IV B-cell non-Hodgkin's lymphoma (B-NHL) with up to 70% FAB L3-type blasts (*n*= 42) in the bone marrow without central nervous system (CNS) disease were treated on the United Kingdom Children Cancer Study Group (UKCCSG) 9002 protocol (identical to the French LMB 84). The median age was 8.3 years. There were 81 boys and 31 girls. According to the extent of the primary disease, patients were sub-staged into three groups: IIIA with unresectable abdominal tumour (*n*= 39); IIIB with abdominal multiorgan involvement (*n*= 57) and IIIX with extra-abdominal primary lymphoma often presenting as pleural effusion (*n*= 16). Univariate and multivariate analyses were carried out to evaluate the prognostic significance of lactate dehydrogenase (LDH) level at diagnosis, the sub-stage and the time to achieve complete remission (CR). With a median follow up of 48 months (range 12–92), the overall and event free survival (EFS) is 87% (95% confidence interval (CI) 79.2–92.1%) and 83.7% (95% CI 76.3–89.2%) respectively. Six patients (5.4%) never achieved CR, of whom one is alive following high-dose therapy. Eight patients (7.1%) relapsed after achieving CR, three are alive after second-line therapy. There were three early toxic deaths (2.7%), mainly from infection, and one late death from a second cancer. There was no significant difference in EFS according to LDH level at diagnosis, the sub-stage or the time to CR. This study confirms the overall good prognosis and low rate of toxic deaths in patients with advanced B-NHL treated with this intensive regimen. No significant difference in EFS according to the sub-stage, the time to achieve CR or LDH level at diagnosis making it difficult to identify a group that should not receive intensive therapy. © 2000 Cancer Research Campaign


*British Journal of Cancer* (2000) **82**(8), 1396–1402
© 2000 Cancer Research Campaign
DOI: 10.1054/ bjoc.1999.1083, available online at http://www.idealibrary.com on IDEAL®

### Improved outcome in children with advanced stage B-cell non-Hodgkin's lymphoma (B-NHL): results of the United Kingdom Children Cancer Study Group (UKCCSG) 9002 protocol

**A Atra[1], JD Imeson[2], R Hobson[2], M Gerrard[3], IM Hann[4], OB Eden[5], RL Carter[1], CR Pinkerton[1] on behalf of the UKCCSG**

[1]Department of Paediatric Oncology, The Royal Marsden Hospital NHS Trust, Downs Road, Sutton, Surrey SM2 5PT, UK; [2]UKCCSG, University of Leicester, Department of Epidemiology and Public Health, 22–28 Princess Road West, Leicester LE1 6TP, UK; [3]Sheffield Children's Hospital NHS Trust, Western Bank, Sheffield S10 2TH, UK; [4]The Hospital For Sick Children, Great Ormond Street, London WC1 3JH, UK; [5]Academic Unit of Paediatric Oncology, Christie Hospital NHS Trust, Manchester M20 9BX, UK

**Summary** From July 1990 to March 1996, 112 children with stage III or IV B-cell non-Hodgkin's lymphoma (B-NHL) with up to 70% FAB L3-type blasts ($n$ = 42) in the bone marrow without central nervous system (CNS) disease were treated on the United Kingdom Children Cancer Study Group (UKCCSG) 9002 protocol (identical to the French LMB 84). The median age was 8.3 years. There were 81 boys and 31 girls. According to the extent of the primary disease, patients were sub-staged into three groups: IIIA with unresectable abdominal tumour ($n$ = 39); IIIB with abdominal multiorgan involvement ($n$ = 57) and IIIX with extra-abdominal primary lymphoma often presenting as pleural effusion ($n$ = 16). Univariate and multivariate analyses were carried out to evaluate the prognostic significance of lactate dehydrogenase (LDH) level at diagnosis, the sub-stage and the time to achieve complete remission (CR). With a median follow up of 48 months (range 12–92), the overall and event free survival (EFS) is 87% (95% confidence interval (CI) 79.2–92.1%) and 83.7% (95% CI 76.3–89.2%) respectively. Six patients (5.4%) never achieved CR, of whom one is alive following high-dose therapy. Eight patients (7.1%) relapsed after achieving CR, three are alive after second-line therapy. There were three early toxic deaths (2.7%), mainly from infection, and one late death from a second cancer. There was no significant difference in EFS according to LDH level at diagnosis, the sub-stage or the time to CR. This study confirms the overall good prognosis and low rate of toxic deaths in patients with advanced B-NHL treated with this intensive regimen. No significant difference in EFS according to the sub-stage, the time to achieve CR or LDH level at diagnosis making it difficult to identify a group that should not receive intensive therapy. © 2000 Cancer Research Campaign

**Keywords**: childhood; B-cell non-Hodgkin's lymphoma; advanced stage III and IV; UKCCSG 9002 protocol

Over the last two decades, various studies have shown consistent improvement in the overall prognosis of children with B-cell non-Hodgkin's lymphoma (B-NHL) (Philip et al, 1982; Al-Attar et al, 1986; Patte et al, 1990). This is attributable to both a reduction in early toxic deaths (Lynch et al, 1977; Allegretta et al, 1985; Miron et al, 1997) and a decrease in relapse rate following an increase in the intensity of systemic and central nervous system (CNS)-directed therapy (Hann et al, 1990; Patte et al, 1991; Cairo et al, 1996). This improvement in the results of treatment has involved patients with all stages of disease (Pinkerton et al, 1991; Patte et al, 1997; Atra et al, 1998). Radiotherapy no longer appears to have a role in the treatment of primary CNS disease (Patte et al, 1986; Bowman et al, 1996), and radical surgery with its potential risks, has become unnecessary (Al-Attar et al, 1989; Miron et al, 1997). Relapse still occurs and is the principle cause of treatment failure. Patients who relapse after first-line chemotherapy can occasionally be salvaged with more intensive chemotherapy with or without megatherapy and stem cell rescue (Philip et al, 1993; Sweetenham et al, 1994).

The United Kingdom Children Cancer Study Group (UKCCSG) 9002 protocol was based on the French LMB-84 and it involved 3 months of treatment with sequential doses of chemotherapy including intrathecal (i.t.) therapy. No radiotherapy was given. We report the results of this regimen in the treatment of children with stage III and IV B-NHL (L3-blasts up to 70% in the bone marrow). The prognostic significance of serum lactate dehydrogenase (LDH) level at diagnosis, tumour substage and time to achieve complete remission (CR) were prospectively analysed in an attempt to identify different risk groups and to facilitate delivery of risk-directed therapy in the future.

### PATIENTS AND METHODS

Children under 18 years with newly diagnosed Murphy stage III or IV B-NHL and up to 70% FAB L3-type blasts in the bone marrow (but with no CNS disease), with B-cell histology (Burkitt's or Burkitt's-like or large cell) and/or phenotype (surface membrane immunoglobulin-positive) were eligible for this protocol. This is the group of patients identified by the LMB group to be of

*Received 20 August 1999*
*Revised 11 November 1999*
*Accepted 16 November 1999*

*Correspondence to:* A Atra

**Table 1** UKCCSG 9002 protocol

| Regimen | Dose | Administration |
|---|---|---|
| COP | | |
| Cyclophosphamide | 300 mg $m^{-2}$ | i.v. bolus day 1 |
| Vincristine | 1 mg $m^{-2}$ | i.v. bolus day 1 |
| Prednisolone | 60 mg $m^{2}$ day | Oral days 1–7 |
| i.t. MTX + HC | | day 1 |
| COPADM 1 | | |
| Vincristine | 2 mg $m^{-2}$ | i.v. bolus day 1 |
| Doxorubicin | 60 mg $m^{-2}$ | i.v. over 6 h day 2 |
| Cyclophosphamide | 250 mg $m^{-2}$ dose | i.v. bolus 12 h days 2–4 (with mesna) |
| High-dose MTX | 3 $m^{-2}$ | i.v. over 3 h day 1 |
| Folinic acid rescue | 15 mg $m^{-2}$ | i.v. from day 2 |
| Prednisolone | 60 mg $m^{-2}$ | oral days 1–5 |
| i.t. MTX+HC | | day 2, 6 |
| COPADM 2 as COPADM1 except | | |
| Vincristine | 2 mg $m^{-2}$ | i.v. bolus day 1, 6 |
| Cyclophosphamide and mesna | 500 mg $m^{-2}$ dose | i.v. bolus 12 hr days 2–4 |
| CYM 1+2 | | |
| Cytarabine | 100 mg $m^{-2}$ day | [illegible]. continuous infusion days 2–6 |
| High-dose MTX | 3 $m^{-2}$ | [illegible]. over 3 h day 1 |
| Folinic acid rescue | 15 mg $m^{-2}$ | i.v. from day 2 |
| i.t. MTX + HC | | day 2 |
| i.t. Cytarabine + HC | | day 7 |
| COPADM 3 AS COPADM 1 (expect that cyclophosphamide is given for 2 days only) | | |

MTX – methotrexate; HC – hydrocortisone; I.T. – intrathecal; I.V. – intravenous

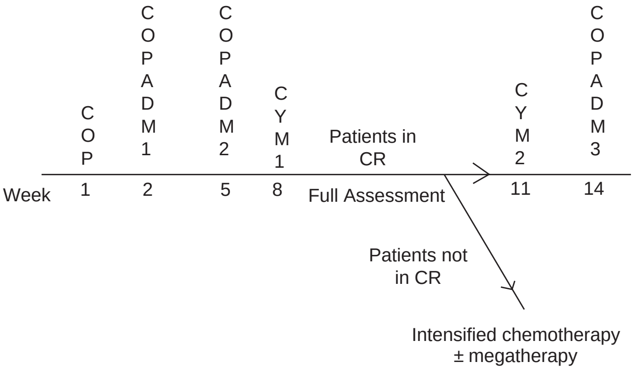

**Figure 1** Schema of the UKCCSG 9002 protocol

'standard risk'. Those with up to 70% L3-type blasts in the bone marrow but no blasts in peripheral blood (who would be defined by the Murphy scheme as B-ALL) are included. Informed consent was obtained from all patients and their parents as appropriate. Staging investigations included bilateral bone marrow aspirates, cerebrospinal fluid (CSF) cell count and cytospins, a chest radiograph and ultrasonography, with or without computerized tomography (CT) scan of the primary tumour. All pathology specimens, biopsies, pleural or ascitic fluid, bone marrows and CSF were reviewed centrally. Surgical attempts were limited to obtain adequate biopsy material for histopathological diagnosis.

One hundred and thirteen consecutively diagnosed and previously untreated patients were entered between July 1990 and March 1996 (one patient was identified after entering the study to be HIV-positive and was subsequently excluded from the analysis). Eighty-one patients were boys and 31 girls. Ages at diagnosis ranged from 2.2 to 17.5 years (median 8.3 years). For the purpose of this study, patients were substaged according to the French Society of Paediatric Oncology (SFOP) classification, into two groups according to the extent of primary disease (Philip et al, 1987). Thirty-nine patients were stage IIIA as defined by the presence of limited but unresectable disease usually localized to the abdomen with or without ascites. Patients with abdominal primary and multi-organ involvement, e.g. kidney, liver or ovary ($n$ = 57), were classified as IIIB. As patients with extra-abdominal primary B-NHL, particularly those with pleural involvement often presenting with effusion, are believed to have a worse outcome (Sandlund et al, 1990); in this study those are classified as IIIX ($n$ = 16). Among the 112 patients, 42 had involvement of the bone marrow at diagnosis with up to 70% L3-blasts. No patient included in this study had CNS disease at diagnosis.

The schema and details of the UKCCSG 9002 protocol are shown in Figure 1 and Table 1 respectively. The first course of treatment consisted of low-dose cyclophosphamide, vincristine and prednisolone (COP) in an attempt to avoid severe drug toxicity, often associated with tumour lysis syndrome at diagnosis. This course could be repeated 1 week later if the clinical condition of the patient remained poor before proceeding with the more intensive treatment of cyclophosphamide, vincristine, prednisolone, doxorubicin and high-dose methotrexate (COPADM). All patients received regular intrathecal (i.t.) hydrocortisone with either methotrexate or cytarabine. Patients were assessed clinically and radiologically to confirm response and exclude progressive disease after COP. Full assessment was done to confirm CR prior to the second block of cytarabine and high-dose methotrexate (CYM2) (Figure 1). Those who achieved CR continued on protocol. In the absence of CR, patients were treated with more intensive chemotherapy as in the UKCCSG 9003 protocol (Atra

© 2000 Cancer Research Campaign

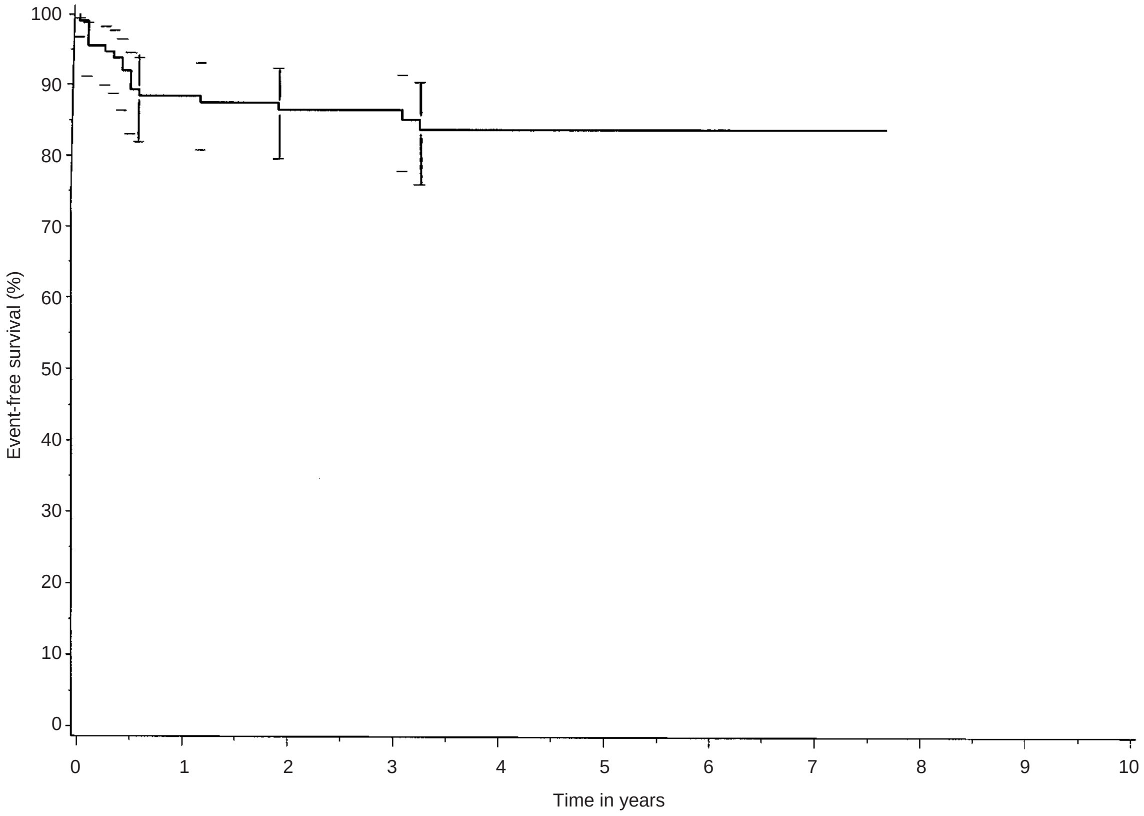

**Figure 2** Event free survival of all patients.

et al, 1998), followed by high-dose chemo/radiotherapy and stem cell rescue. Patients were formally assessed at the end of treatment to confirm remission with 1–2 monthly review for the first year after the end of treatment. The follow-up was by clinical examination and radiological imaging was only performed if there was clinical concern.

All patients received appropriate supportive care immediately after diagnosis. Antimicrobials, blood and platelet transfusions and total parental nutrition (TPN) were used as indicated. During induction therapy hyper-hydration was given using dextrose–saline 3 l $m^{-2}$ per day and allopurinol 10 mg $kg^{-1}$ $day^{-1}$. Dialysis (peritoneal or haemodialysis) was performed according to the clinical situation. No patient received prophylactic granulocyte colony-stimulating factor (G-CSF).

### Statistical methods

The main end point used to evaluate the protocol treatment was event-free survival (EFS). This was defined as the length of time from diagnosis to the earliest detection of relapse/progression or death from any cause. Surviving relapse-free patients were censored at the date of the last clinical follow-up and survival curves were calculated by standard methods (Kaplan and Meier, 1958; Rothman, 1978). Univariate analysis was carried out using the log-rank test (Peto et al, 1977) to evaluate the differences in prognosis between patients in relation to the sub-stage – IIIA vs IIIB vs IIIX; LDH level at diagnosis twice or more vs less than twice the institutional upper limit normal (ULN) and the achievement of CR within less than 28 versus 28 days or more. Multivariate analysis was carried out including all these factors together to test for independent effects (SAS, 1991).

### RESULTS

Six patients (5.4%) never achieved CR. All died 1–6 months from diagnosis (median 3.6 months) except one who continued to have residual lymphoma (biopsy proven) at the primary site. He went into CR after high-dose chemotherapy/radiotherapy and autologous bone marrow rescue and is alive 2 years from diagnosis.

At the time of analysis, eight patients (7.1%) have relapsed after achieving CR. Sites of relapse were combined primary site and bone marrow ($n$ = 2), bone marrow ($n$ = 2), primary site ($n$ = 3) and one patient relapsed in the bone marrow, primary site and CNS. Five of these eight patients died 6–14 months from diagnosis (median 9.2 months) despite receiving subsequent more intensive chemotherapy ($n$ = 4) or high-dose chemo/radiotherapy with autologous stem cell rescue ($n$ = 1). Three are alive in CR after receiving further intensive chemotherapy using the UKCCSG 9003 protocol or CHOP, followed by autologous stem rescue ($n$ = 2) or matched unrelated bone marrow ($n$ = 1), 2–6 years from diagnosis (median 3.6 years).

### Toxicity

Three patients (2.7%) died of early toxicity. Causes of death were disseminated aspergillosis at 35 days ($n$ = 1), sepsis with post-mortem evidence of anthracycline-induced cardiomyopathy at 30 days ($n$ = 1) and peritonitis with renal cortical necrosis and

© 2000 Cancer Research Campaign

**Table 2** Results of univariate and multivariate analyses of EFS

| | Log rank *P*-value Univariate | Relative risk multivariate | *P*-value multivariate |
|---|---|---|---|
| LDH (≥vs<2 ULN) | 0.75 | 0.85 | 0.76 |
| CR (>28 days vs <28 days) | 0.47 | 1.33 | 0.71 |
| Substage (IIIA vs IIIB) | 0.82 | 0.56 | 0.64 (0.38) |
| (IIIX vs IIIB) | | 0.71 | (0.67) |

ULN – upper limit of normal

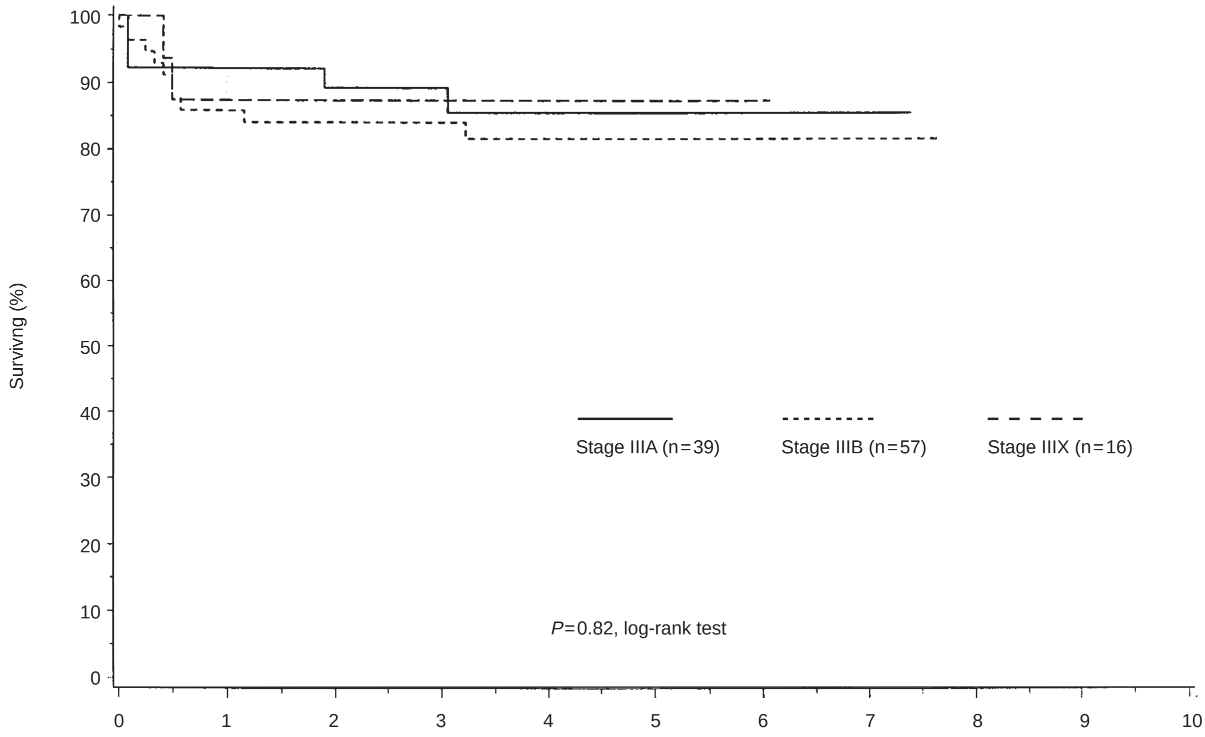

**Figure 3** Event free survival in relation to the substage congestive cardiac failure 52 days from diagnosis ($n = 1$). There was one late death in a patient who developed chronic myeloid leukaemia 21 months from diagnosis. He was treated with three intensive blocks of AML-type chemotherapy with good initial response, but no further treatment was given because of severe cardiac toxicity. He subsequently progressed to develop an acute myeloblastic crisis and died 15 months later. Acute mucosal and myelotoxicity was significant, but managable as previously reported (Patte et al, 1991).

### Survival

With a median follow-up of 48 months (range 12–92), the overall survival and EFS are 87% (95% confidence interval (CI) 79.2–92.1%) and 83.7% (95% CI 76.3–89.2%) respectively (Figure 2). Results of univariate and multivariate analyses of EFS are shown in Table 2.

LDH levels at diagnosis were available in 79 patients with a range of 154–7575 iu $l^{-1}$ (median 1118). The median time to achieve CR was 70 days (range 7–181). The overall survival and EFS are high and differences in EFS according to the three sub-stages identified, LDH level or the time to achieve CR are small at 4 years from diagnosis (Figures 3, 4 and 5). The multivariate analysis does not appear to change the conclusions from the univariate analysis.

### DISCUSSION

The consistent improvement in the prognosis of children with B-NHL has been mainly due to the use of higher dose systemic chemotherapy, frequent i.t. therapy and the reduction in early toxic death by avoiding unnecessary surgery coupled with aggressive treatment of biochemical and infectious complications soon after diagnosis or during induction (Lynch et al, 1977; Hann et al, 1990; Patte et al, 1991; Cairo et al, 1996; Miron et al, 1997). In the last decade the emphasis has been on trying to find prognostic factors that help to stratify patients and to deliver treatment according to risk group. The SFOP classification subdivided patients with stage

© 2000 Cancer Research Campaign

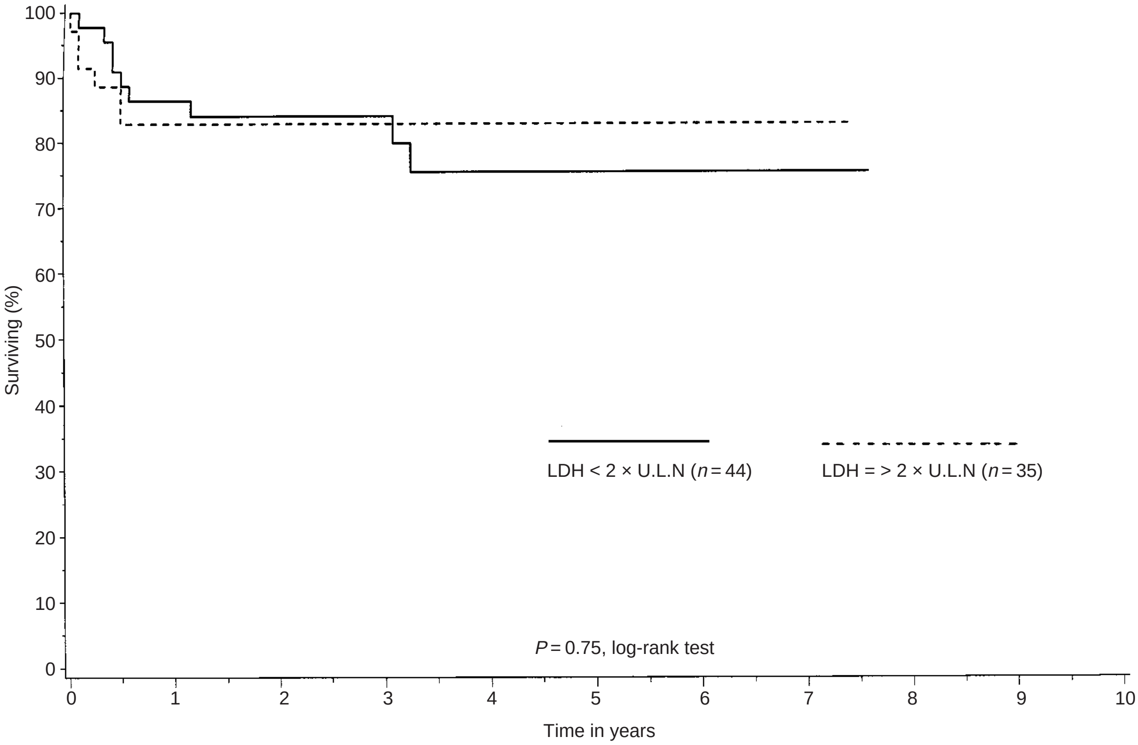

**Figure 4** Event free survival in relation to serum LDH level at diagnosis

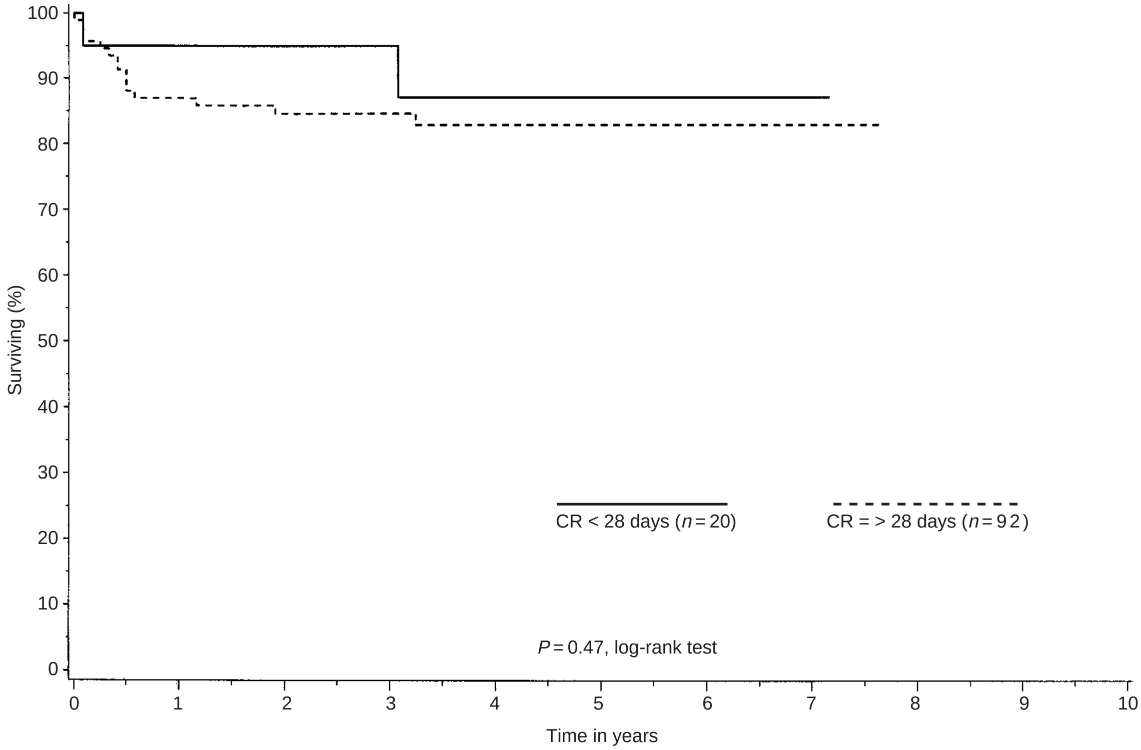

**Figure 5** Event free survival in relation to time to achieve CR

III disease into two main groups, namely IIIA and IIIB, according to the extent of primary disease (Philip et al, 1987). The German BFM group used a similar approach by dividing patients with stage III B-NHL into two risk groups based on LDH levels less than or more than 500 u $l^{-1}$ (Reiter et al, 1995). Children with LDH less than 500 (R2 Regimen) and those with LDH more than 500 u

*© 2000 Cancer Research Campaign*

l[-1] (R3 Regimen) had EFS at 5 years of 95% and 68% respectively (Reiter et al, 1997). The EFS for all stage III patients improved from 73% in the BFM 86 study to 90% in the BFM 90 study where the treatment was intensified.

A randomized prospective study by the CCG (CCG-503) compared two chemotherapy regimens (COMP vs D-COMP) in children with disseminated non-lymphoblastic NHL and no bone marrow or CNS disease. This showed a 2-year EFS of 63%. Those with bone marrow or CNS involvement were electively treated with the D-COMP regimen and had a 2-year EFS of 48% (Chilcote et al, 1991). Children with LDH of 500 or more, bone marrow or CNS involvement and the presence of thoracic lymph node enlargement had a poorer outcome.

The CCG also compared the results of treatment of children with B-NHL and bone marrow or CNS involvement in a randomized study using the LMB-89 and CCG hybrid designated the 'Orange' protocol. There was no significant difference in EFS between the two groups, but longer duration of hospitalization and more toxicity was recorded with the former (Cairo et al, 1996). Poorer outcome was associated with serum LDH greater than twice institutional norms and CNS involvement.

The POG-86 Study involved 133 children with B-ALL ($n$ = 74) and stage IV B-NHL ($n$ = 59) treated with intensified systemic and i.t. chemotherapy including low-dose continuous infusion cytarabine. Sixty-three patients had abdominal disease and 36 had CNS involvement at diagnosis. The EFS for children with B-ALL and stage IV B-NHL was 65% and 79% respectively (Bowman et al, 1996).

In the UKCCSG NHL-86 study, children with Murphy stage III B-NHL were treated with standard dose chemotherapy ($n$ = 28) including 15 with extensive multiorgan disease or a more intensive regimen (MACHO) ($n$ = 16), 14 with multiorgan involvement. The EFS was 76% with a lower relapse rate in the latter group (6% vs 21%) (Hann et al, 1990; Pinkerton et al, 1991).

In the early 1990s, the UKCCSG adopted the French LMB 84 protocol for the treatment of children with advanced stage B-NHL with the aim of both reproducing the excellent SFOP results and to study the prognostic significance of LDH level at diagnosis, substage and the time to achieve CR. In addition to the previous sub-stages IIIA and IIIB, patients with extra abdominal disease were classified as IIIX. Univariate and multivariate analyses showed no evidence of prognostic value for substage, time to achieve CR or LDH. The limited number of patients with known LDH might have contributed to the lack of prognostic significance of LDH level. Another aim of the study was to attempt to reduce early toxic death rate by preventing tumour lysis syndrome during early induction and aggressive treatment of infectious and renal complications, which had appeared to be higher in the previous UKCCSG experience (Hann et al, 1990; Pinkerton et al, 1991), than with the LMB studies (Philip et al, 1987). Three patients (2.7%) died due to treatment-related toxicity, compared with reported rates of 6–8% in previous UKCCSG studies. This reduction is encouraging but efforts need to be made to reduce it even further, e.g. the use of urate oxidase (uricozyme). The relapse rate of 7.1% in our study is comparable to that in SFOP studies, and CNS relapse of less than 1% remains low despite the absence of cranial radiotherapy. Three of the relapsed patients in this study were salvaged with more intensive chemotherapy, followed by high-dose chemo/radiotherapy and a stem cell rescue confirming previous reports that relapsed patients after less intensive first-line therapy may be cured with more intensive chemotherapy (Philip et al, 1993; Sweetenham et al, 1994).

We conclude that with a short LMB type regimen of moderately intensive multi-agent chemotherapy, patients with a stage III and IV (blasts less than 70%) B-NHL have an excellent prognosis and as therapy has become more intensive and effective, the significance of various prognostic factors disappear. The priority in this group is to try and reduce both early and late morbidity. This is the aim of the current UK, French and US collaboration (FAB LMB 96) where reduction in both dose intensity and duration are evaluated in a randomized study. It is apparent that, with such effective chemotherapy, no clear prognostic subgroups can be identified at presentation. Something could emerge in the eventual analysis of FAB LMB 96 where treatment has been reduced for some patients. It is important that new approaches are found for relapsed patients, although few in number, the salvage rate for these patients remains relatively poor.

### REFERENCES

Al-Attar A, Pritchard J, Al-Saleem T, Al-Naimi M, Alash N and Atra A (1986) Intensive chemotherapy for non-localised Burkitt's lymphoma. *Arch Dis Child* **61**: 1013–1019

Al-Attar A, Atra A, Al-Bagdadi R, Al-Naimi M, Al-Saleem T and Pritchard J (1989) 'Debulking' surgery is unnecessary in advanced abdominal Burkitt lymphoma in Iraq. *Br J Cancer* **59**: 610–612

Allegretta GJ, Weismann SJ and Altman AJ (1985) Metabolic and space-occupying consequences of cancer and cancer treatment. *Ped Clin N Am* **32**: 601

Atra A, Gerrard M, Hobson R, Imeson JD, Ashley S and Pinkerton CR on behalf of the UKCCSG (1998) Improved cure rate in children with B-cell acute lymphoblastic leukaemia (B-ALL) and Stage IV B-cell non-Hodgkin's lymphoma (B-NHL) – results of the UKCCSG 9003 protocol. *Br J Cancer* **77**: 2281–2285

Bowman WP, Shuster JJ, Cook B, Griffin T, Behm F, Pullen J, Link M, Head D, Carroll A, Berard C and Murphy S (1996) Improved survival for children with B-cell acute lymphoblastic leukaemia and stage IV small noncleaved-cell lymphoma: a pediatric oncology study group. *J Clin Oncol* **14**: 1252–1261

Cairo M, Krailo M, Morse M, Hutchinson R, Harris R, Kjeldsberg C, Kadin M, Radel E, Steinherz L and Meadow A (1996) Disseminated non-lymphoblastic non-Hodgkin's lymphoma (DNLHL) of childhood: a randomised phase II trial of short intensive treatment (abstract 093). *Ann Oncol* **7**: 20

Chilcote RR, Krailo M, Kjeldsberg C, Kadin M, Steinherz P, Coccia P, Morse M, Reaman G and Hammond G (1991) Daunomycin plus COMP therapy in childhood non-lymphoblastic lymphomas. *Proc Am Soc Clin Oncol* **10**: 289 (abstract)

Hann IM, Eden OB, Barnes J and Pinkerton CR (1990) MACHO chemotherapy for Stage IV B-cell lymphoma and B-cell acute lymphoblastic leukaemia of childhood. *Br J Haem* **76**: 359–364

Kaplan EL and Meier P (1958) Non-parametric estimation from incomplete observations. *J Am Stat Ass* **53**: 457–481

Lynch RE, Kjellstrand CM and Coccia PF (1977) Renal and metabolic complications of childhood non-Hodgkin's lymphoma. *Semin Oncol* **4**: 235

Miron I, Frappaz D, Brunat-Mentingy M, Combaret V, Buclon M, Bouffet E, Thiesse P, Ragg S, Bailly C and Philip T (1997) Initial management of advanced Burkitt lymphoma in children: Is there still a place for surgery? *Ped Hem Oncol* **14**: 555–561

Patte C, Philip T, Rodary C, Bernrd A, Zuker JM, Bernard JL, Robert A, Railland X, Benz-Lemoine E and Demeocq F (1986) Improved survival rate in children with Stage III and IV B-cell non-Hodgkin's lymphoma and leukaemia using multi-agent chemotherapy: results of a study of 114 children from the French Pediatric Oncology Society. *J Clin Oncol* **4**: 1219–1226

Patte C, Leverger G, Perel Y, Rubie H, Otten J, Nelken B, Gentet JC, Lumley L De, Berendt H and Brugleres L, for the SFOP (1990) Updated results of the LMB 86 protocol of the French Pediatric Oncology Society (SFOP) for B-cell non-Hodgkin's lymphomas 9B-NHL with CNS involvement (CNS+) and B-ALL (abstract 22). *Med Ped Oncol* **18**: 397

Patte C, Philip T, Rodary C, Zuker JM, Behrendt H, Gentet JC, Lamagnere JB, Otten J, Dufillot D, Pein F, Caillou B and Lemerle J (1991) High survival rate in advanced stage B cell lymphoma and leukaemias without CNS involvement with a short intensive polychemotherapy: results from the French Pediatric Oncology Society of a randomised trial of 216 children. *J Clin Oncol* **9**: 123–132

© 2000 Cancer Research Campaign 

Patte C, Michon J, Berendt H, Leverger G, Frappaz D, Robert A, Mechinaud F, Bertrand Y, Perel Y, Coze C and Nelken B on behalf of the SFOP (1997) Updated results of the LMB 89 protocol of the SFOP (French Pediatric Oncology Society) for childhood B-cell lymphoma and leukaemia (ALL) *Med Ped Oncol* **29**: 358 (abstr SIOP)

Peto R, Pike MC, Armitage P, Breslow NE, Cox DR, Howard SV, Mantel N, McPherson K, Peto J and Smith PG (1977) Design and analysis of randomised clinical trials requiring prolonged observation of each patient. II Analysis and examples. *Br J Cancer* **35**: 1–39

Philip T, Lenoir GM, Bryon PA, Souillet G, Philippe N, Freycon F and Brunat-Mentigny M (1982) Burkitt type lymphoma in France among non-Hodgkin malignant lymphomas in Caucasian children. *Br J Cancer* **45**: 670–678

Philip T, Pinkerton CR, Biron P, Ladjadj Y, Bouffet E, Souillet G, Philippe N, Frappaz D, Freycon F, Chauvin F and Brunat-Mentignj M (1987). Effective multi-agent chemotherapy in children with advanced B-cell lymphoma: who remains the high risk patient? *Br J Haematol* **65**: 159–164

Philip T, Hartmann O, Pinkerton R, Zuker JM, Gentet JC, Lamagnere JP, Berhendt H, Perel Y, Otten J, Lutz P, Rodary C, Caillou B, Bayle C, Chauvin F and Patte C (1993) Curability of relapsed childhood B-cell non-Hodgkin's lymphoma after intensive first line therapy: A report from the Societe Francaise d'Oncologie Pediatrique. *Blood* **81**: 2003–2006

Pinkerton CR, Hann I, Eden OB, Gerrard M, Berry J and Mott MG on behalf of the United Kingdom Children's Cancer Study Group (1991) Outcome in Stage III non-Hodgkin's lymphoma in children (UKCCSG study NHL 86) – How much treatment is needed? *Br J Cancer* **64**: 583–587

Reiter A, Schrappe M, Parwaresch R, Henze G, Muller-Weihrich S, Sauter S, Sykora KW, Ludwig WD, Gander H and Riehm H (1995) Non-Hodgkin's lymphomas of childhood and adolescence: results of a treatment stratified from biological subtypes and stage – a report of the Berlin-Frankfurt-Munster Group. *J Clin Oncol* **13**: 359–372

Reiter A, Schrappe M, Tiemann M, Yakisan E, Zimmermann M, Ebell W, Meyer U, Schirg E, Sykora KW, Pluss HJ, Mann G, Henze G, St. Muller-Weihrich, Gadner H, Parwaresch R, Riehm H, for the BFM group, Medizinische Hochschule Hannover, Germany (1997) Treatment results for the B-cell lymphomas and acute B-cell leukaemia in the German-Austrian-Swiss study NHL-BFM 90. A report of the BFM group. Risk group definition, treatment strategy and preliminary results for B-cell neoplasias in trial NHL-BFM 90. *Med Ped Oncol* **29**: 358 (abstr)

Rothman KJ (1978) Estimation of confidence limits for the cumulative probability of survival in life table analysis. *J Chronic Dis* **31**: 557–560

Sandlund J, Crist W, Fairclough D, Berard C and Pui CH (1990) Pleural effusion confers a worse treatment outcome for children with stage III abdominal small noncleaved cell non-Hodgkin's lymphoma. *Proc ASCO* **9**: 1065 (abstract)

SAS (1991): The PHREG procedure. SAS Institute Inc: Cary, NC

Sweetenham JW, Liberti G, Pearce R, Taghipour G, Santini G and Goldstone AH (1994) High-dose therapy and autologous bone marrow transplantation for adult patients with lymphoblastic lymphoma: results of the European group for bone marrow transplantation. *J Clin Oncol* **12**: 1358–1365

 *© 2000 Cancer Research Campaign*

## References

[PDF_00471] Al-Attar A., Pritchard J., Al-Saleem T., Al-Naimi M., Alash N., Attra A. (1986). Intensive chemotherapy for non-localised Burkitt's lymphoma.. Arch Dis Child.

[PDF_00477] Allegretta G. J., Weisman S. J., Altman A. J. (1985). Oncologic emergencies I. Metabolic and space-occupying consequences of cancer and cancer treatment.. Pediatr Clin North Am.

[PDF_00479] Atra A., Gerrard M., Hobson R., Imeson J. D., Ashley S., Pinkerton C. R. (1998). Improved cure rate in children with B-cell acute lymphoblastic leukaemia (B-ALL) and stage IV B-cell non-Hodgkin's lymphoma (B-NHL)--results of the UKCCSG 9003 protocol.. Br J Cancer.

[PDF_00484] Bowman W. P., Shuster J. J., Cook B., Griffin T., Behm F., Pullen J., Link M., Head D., Carroll A., Berard C. (1996). Improved survival for children with B-cell acute lymphoblastic leukemia and stage IV small noncleaved-cell lymphoma: a pediatric oncology group study.. J Clin Oncol.

[PDF_00496] Hann I. M., Eden O. B., Barnes J., Pinkerton C. R. (1990). 'MACHO' chemotherapy for stage IV B cell lymphoma and B cell acute lymphoblastic leukaemia of childhood. United Kingdom Children's Cancer Study Group (UKCCSG).. Br J Haematol.

[PDF_00503] Miron I., Frappaz D., Brunat-Mentigny M., Combaret V., Buclon M., Bouffet E., Thiesse P., Ragg S., Bailly C., Philip T. (1997). Initial management of advanced Burkitt lymphoma in children: is there still a place for surgery?. Pediatr Hematol Oncol.

[PDF_00507] Patte C., Philip T., Rodary C., Bernard A., Zucker J. M., Bernard J. L., Robert A., Rialland X., Benz-Lemoine E., Demeocq F. (1986). Improved survival rate in children with stage III and IV B cell non-Hodgkin's lymphoma and leukemia using multi-agent chemotherapy: results of a study of 114 children from the French Pediatric Oncology Society.. J Clin Oncol.

[PDF_00517] Patte C., Philip T., Rodary C., Zucker J. M., Behrendt H., Gentet J. C., Lamagnère J. P., Otten J., Dufillot D., Pein F. (1991). High survival rate in advanced-stage B-cell lymphomas and leukemias without CNS involvement with a short intensive polychemotherapy: results from the French Pediatric Oncology Society of a randomized trial of 216 children.. J Clin Oncol.

[PDF_00532] Peto R., Pike M. C., Armitage P., Breslow N. E., Cox D. R., Howard S. V., Mantel N., McPherson K., Peto J., Smith P. G. (1977). Design and analysis of randomized clinical trials requiring prolonged observation of each patient. II. analysis and examples.. Br J Cancer.

[PDF_00543] Philip T., Hartmann O., Pinkerton R., Zucker J. M., Gentet J. C., Lamagnere J. P., Berhendt H., Perel Y., Otten J., Lutz P. (1993). Curability of relapsed childhood B-cell non-Hodgkin's lymphoma after intensive first line therapy: a report from the Société Française d'Oncologie Pédiatrique.. Blood.

[PDF_00537] Philip T., Lenoir G. M., Bryon P. A., Gerard-Marchant R., Souillet G., Philippe N., Freycon F., Brunat-Mentigny M. (1982). Burkitt-type lymphoma in France among non-Hodgkin malignant lymphomas in Caucasian children.. Br J Cancer.

[PDF_00539] Philip T., Pinkerton R., Biron P., Ladjadj Y., Bouffet E., Souillet G., Philippe N., Frappaz D., Freycon F., Chauvin F. (1987). Effective multiagent chemotherapy in children with advanced B-cell lymphoma: who remains the high risk patient?. Br J Haematol.

[PDF_00548] Pinkerton C. R., Hann I., Eden O. B., Gerrard M., Berry J., Mott M. G. (1991). Outcome in stage III non-Hodgkin's lymphoma in children (UKCCSG study NHL 86)--how much treatment is needed? United Kingdom Children's Cancer Study Group.. Br J Cancer.

[PDF_00552] Reiter A., Schrappe M., Parwaresch R., Henze G., Müller-Weihrich S., Sauter S., Sykora K. W., Ludwig W. D., Gadner H., Riehm H. (1995). Non-Hodgkin's lymphomas of childhood and adolescence: results of a treatment stratified for biologic subtypes and stage--a report of the Berlin-Frankfurt-Münster Group.. J Clin Oncol.

[PDF_00565] Rothman K. J. (1978). Estimation of confidence limits for the cumulative probability of survival in life table analysis.. J Chronic Dis.

[PDF_00571] Sweetenham J. W., Liberti G., Pearce R., Taghipour G., Santini G., Goldstone A. H. (1994). High-dose therapy and autologous bone marrow transplantation for adult patients with lymphoblastic lymphoma: results of the European Group for Bone Marrow Transplantation.. J Clin Oncol.

[PDF_00474] al-Attar A., Attra A., al-Bagdadi R., al-Naimi M., al-Saleem T., Pritchard J. (1989). 'Debulking' surgery is unnecessary in advanced abdominal Burkitt lymphoma in Iraq.. Br J Cancer.

